# Comparative transcriptome analyses reveal two distinct transcriptional modules associated with pollen shedding time in pine

**DOI:** 10.1186/s12864-020-06880-9

**Published:** 2020-07-22

**Authors:** Jing-Jing Ma, Shuang-Wei Liu, Fang-Xu Han, Wei Li, Yue Li, Shi-Hui Niu

**Affiliations:** Beijing Advanced Innovation Center for Tree Breeding by Molecular Design, National Engineering Laboratory for Tree Breeding, College of Biological Sciences and Technology, Beijing Forestry University, Beijing, 100083 People’s Republic of China

**Keywords:** *Pinus tabuliformis* Carr, Conifer, Temperature, Comparative transcriptome, Pollen shedding time, Flowering time

## Abstract

**Background:**

Seasonal flowering time is an ecologically and economically important trait in temperate trees. Previous studies have shown that temperature in many tree species plays a pivotal role in regulating flowering time. However, genetic control of flowering time is not synchronised in different individual trees under comparable temperature conditions, the underlying molecular mechanism is mainly to be investigated.

**Results:**

In the present study, we analysed the transcript abundance in male cones and needles from six early pollen-shedding trees (EPs) and six neighbouring late pollen-shedding trees (LPs) in *Pinus tabuliformis* at three consecutive time points in early spring. We found that the EPs and LPs had distinct preferred transcriptional modules in their male cones and, interestingly, the expression pattern was also consistently maintained in needles even during the winter dormancy period. Additionally, the preferred pattern in EPs was also adopted by other fast-growing tissues, such as elongating new shoots. Enhancement of nucleic acid synthesis and stress resistance pathways under cold conditions can facilitate rapid growth and maintain higher transcriptional activity.

**Conclusions:**

During the cold winter and early spring seasons, the EPs were more sensitive to relatively warmer temperatures and showed higher transcriptomic activity than the LPs, indicating that EPs required less heat accumulation for pollen shedding than LPs. These results provided a transcriptomic-wide understanding of the temporal regulation of pollen shedding in pines.

## Background

In seed plants, flowering is an important biological activity for survival at the right time to take advantage of favourable environmental conditions [1]. Flowering time in cultivated crops is also an ecologically and economically important trait, as flowering time is closely associated with crop yield. In *Arabidopsis,* as a model plant, considerable progress had been reported, with important agricultural crops, which had provided a in depth understanding of flowering time regulation in annual plants [2, 3]. However, the understanding of flowering time regulation in most perennial tree species is still limited and need to be investigated. Furthermore, as temperate zone woody plants display annual cycles of growth behaviour, the term “flowering time” generally has two different meanings in temperate trees: the first refers to the first flowering in the multi-year phase transition from vegetative growth to reproductive growth and the formation of reproductive organs, and the second refers to repeated seasonal flowering and the annual opening of inflorescence buds which developed during the previous growing season in reproductively mature trees. The latter issue has long been ignored in studies on model plants, because it only takes 5 days from emergence of the inflorescence to the flowering in *Arabidopsis* [4]; however, in higher woody perennial trees it often takes several months, e.g. 10 months in *Pinus tabuliformis*.

The reported model suggests that temperature, rather than photoperiod, is the critical factor for bud burst in trees [5]. This is evident that dormancy release in the spring in conifers is correlated with temperatures reaching a certain threshold [6–8]. Temperature also appeared to be a key regulator for seasonal flowering time in trees. A phenomenon has been observed in the northern hemisphere whereby the flowering time of trees is much earlier at warmer northern sites than at cooler southern sites. Climatic warming has been noted to alter the onset of flowering in many woody plants significantly [9], and several models based on heat accumulation have accurately predicted the flowering dates of different trees [10, 11]. Indeed, male cone (microsporangiate strobili)  bagging treatments can advance the date of pollen shedding in pines [12]. In natural populations of temperate trees, however, the flowering time is not synchronized. Some trees may require more heat build-up to induce flowering, while others may require less. We have observed previously the flowering times of *P. tabuliformis* in a seed orchard for over 12 years for a pine breeding program [13]. We noticed that although grown under the same temperature conditions, some clones consistently shed pollen earlier than other neighbouring trees, indicating that pollen shedding time is under strict genetic control, and temperature sensitivity likely varies among individual trees.

To determine whether key regulators as transcriptional factors or transcriptional modules are differentially expressed between early pollen-shedding trees (EPs) and late pollen-shedding trees (LPs) in *P. tabuliformis*, we analysed the transcript abundance of six EPs and six LPs in male cones and needles at three consecutive time points near the bud burst date in early spring. The needles were also been analysed because during the winter pines do not defoliate, and the naked needles may be more sensitive to environmental factors /stress than scaly, coated male cone buds. Our results provide insight into the stable expression patterns signatures of pollen shedding time regulation at the genome-wide level in pine species.

## Results

### Time-course RNA-sequencing of male cones and needles between EPs and LPs

**Fig. 1:**
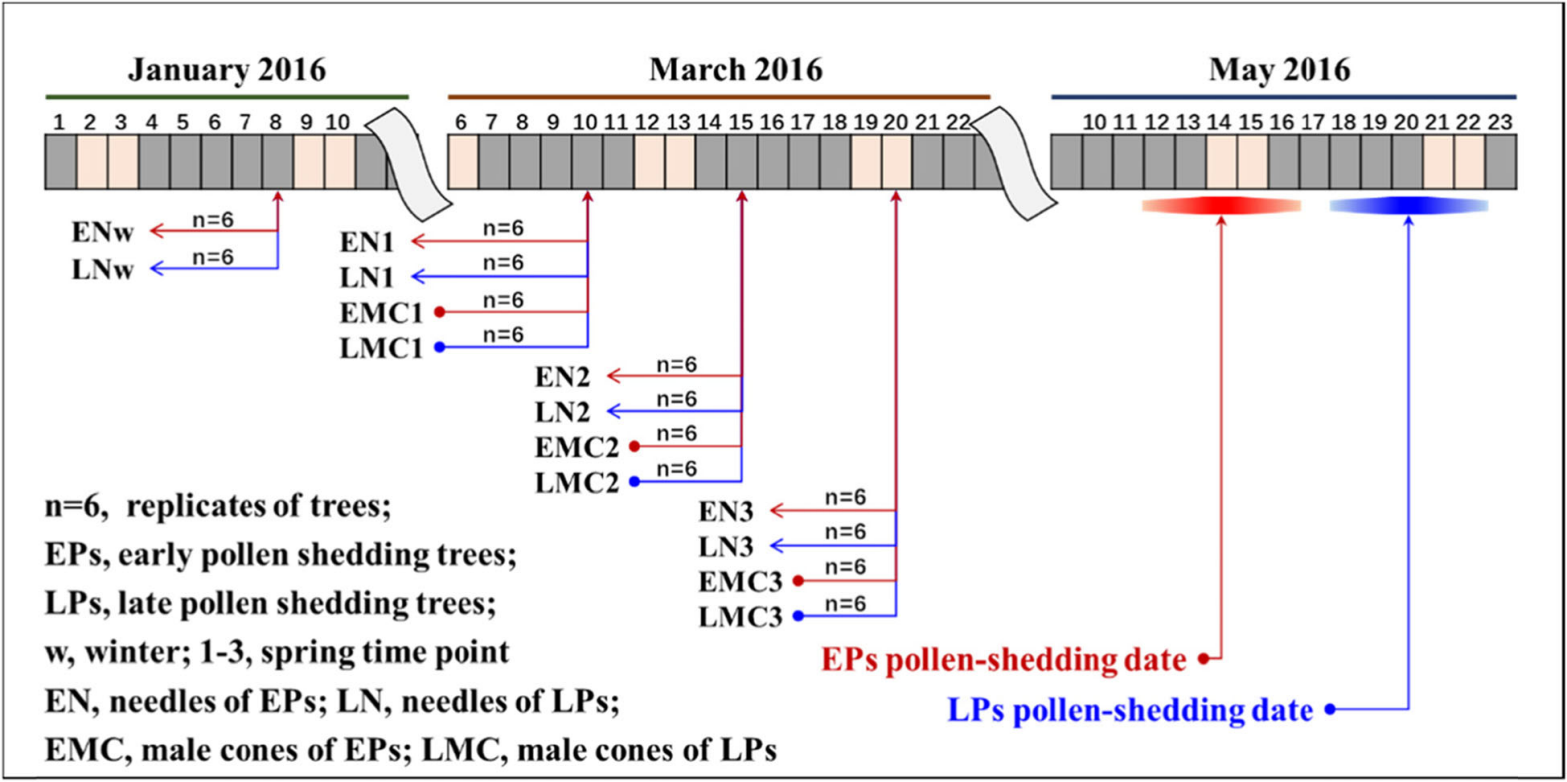
Sampling times and pollen shedding dates of EPs and LPs for RNA-Seq analysis is this study

The annual pollen-shedding dates were recorded from 217 different *P. tabuliformis* clones from May 10–30 in the seed orchard located in Hebei, China. However, for each tree, pollen shedding time generally persisted only for 2–3 days (Fig. 1). We previously observed the flowering time of *P. tabuliformis* over 12 years [13]. Several neighbouring EPs and LPs were selected, such that the EPs always shed pollen earlier than the LPs from 2011 to 2016. In early spring, the axillary bud break was generally visible when the minimum temperature was close to or above 0 °C, which indicates the onset of fresh growth cycle. To validate whether the male cones were already developed differently before the visible bud break, or whether the male cones from EPs and LPs developed differently during early spring, we analysed the transcriptomic profiles of male cones from six EPs and six LPs closely planted within area of 100 square meter. A total of 84 samples including 36 male cones and 48 needles were collected in 2016 and analysed by RNA-seq (Fig. 1).

### Male cones of EPs and LPs do not exhibit strong genomic signatures of significant developmental differences in early spring

**Fig. 2:**
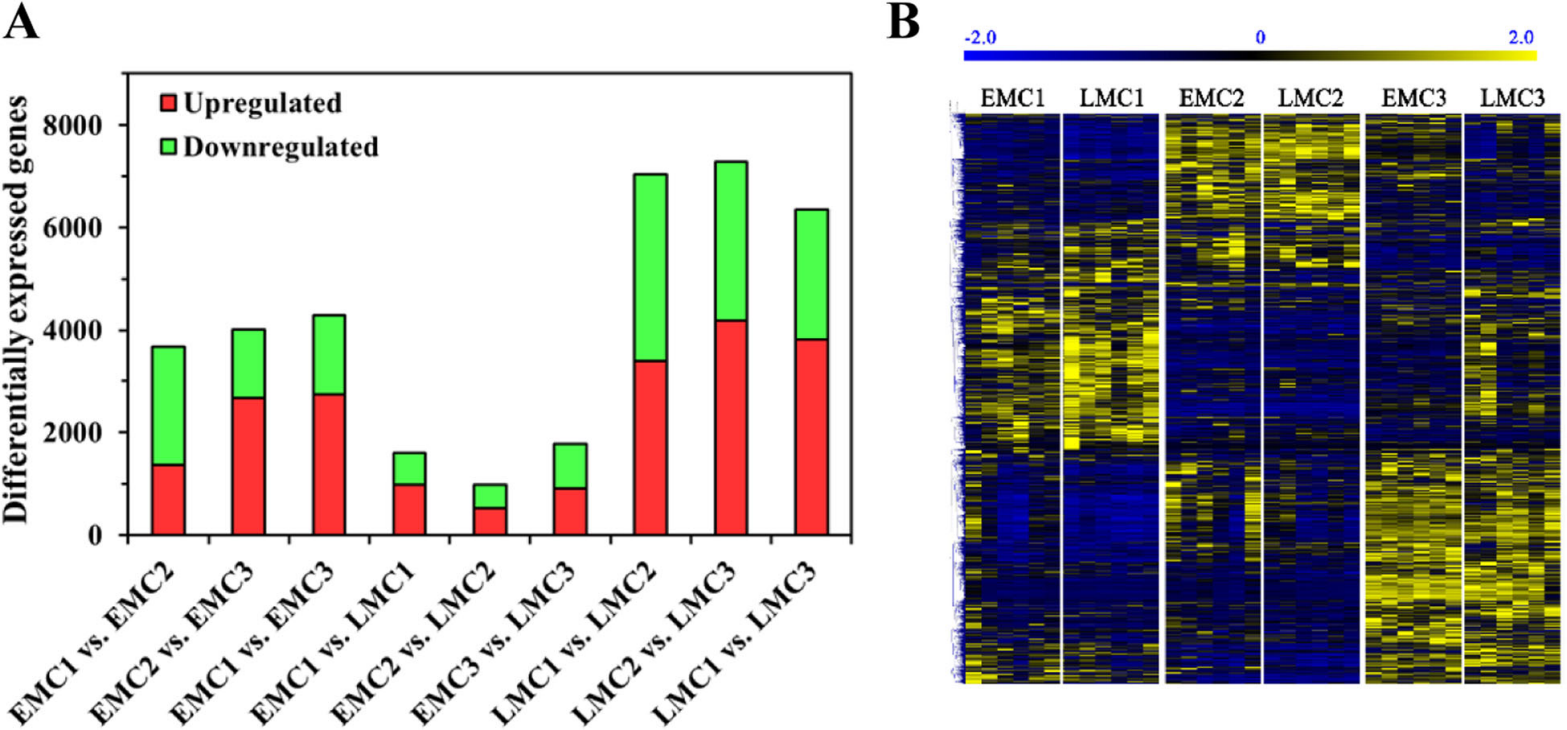
Differentially expressed genes between male cones of EPs and LPs. **a** The number of differentially expressed genes among different sample groups. EMCs and LMCs indicate male cones of EPs and LPs, respectively. The numerical order indicates different sampling times (Fig. 1). Using a threshold value of *P* < 0.01 for filtering differentially expressed genes, a total of 8342, 13405, and 3484 genes were identified between EMCs and LMCs at different time points, and between EMCs and LMCs at the same time point. **b** The 6898 genes that were significantly differentially expressed between the two sample groups (*P* < 0.01, effect size ≥1) are shown in the heat map. Differences were mainly present among sample groups at different time points rather than between EPs and LPs

We identified a total of 52,430 transcripts that were expressed in male cones (in at least one sample group, transcripts per million [TPM] > 1). Surprisingly, we found larger transcriptomic shifts during male cone development over a 10-day period in early spring. More than 15.9 and 25.6% of expressed genes in EP male cones (EMCs) and LP male cones (LMCs) exhibited significant expressional changes between at least two time points (*P* < 0.01). Majority of the genes showed differential expression pattern in LMCs as compared to EMCs (Fig. 2a). Interestingly, the number of differentially expressed genes between EMCs and LMCs at any time point was significantly lower than the number of genes differentially expressed between different time points in EMC or LMC samples (Fig. 2a). In total, the number of intra-group differentially expressed genes in LMCs and EMCs were 3.8-fold and 2.4-fold higherthan the number of differentially expressed genes between LMCs and EMCs (Fig. 2a). As shown in the heat map, the EMCs and LMCs did not exhibit strong genomic signatures of significant developmental differences in early spring (Fig. 2b), at least the difference between EMCs and LMCs was smaller than that caused by 5 to 10 days of development. These results suggest that the EPs may require less time to initiate pollen shedding than LPs, rather than initiating earlier growth in the spring.

### Gene expression patterns of *MADS-box* transcription factors, *FT/TFL*1-like, *LEAFY/NEEDLY* (*LFY/NDLY*), and *EBB1* genes in male cones of EPs and LPs

**Fig. 3:**
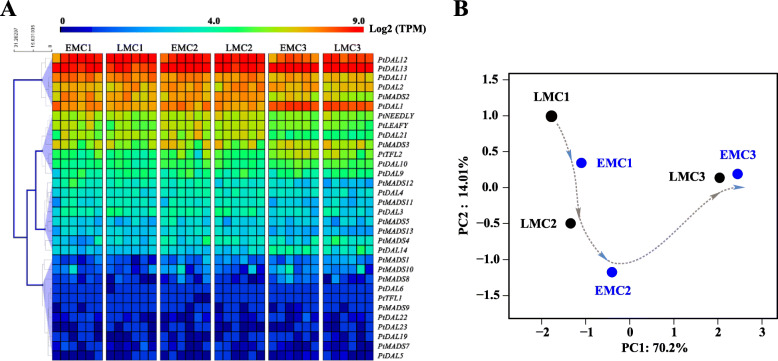
Expression profiles of *MADS-box* genes, *FT/TFL1*-like, and *LEAFY/NEEDLY* in EMCs and LMCs. **a** Heat map and cluster analysis of *MADS-box* genes, *FT/TFL1*-like, and *LEAFY/NEEDLY* in EMCs and LMCs. The data from six biological replicates are shown individually. TPM, transcripts per million. **b** Principal component analysis (PCA) based on the mean expression levels of each gene: comparison between six biological replicates. Each symbol represents a sample group (*n* = 6)

*MADS-box* genes, *FT/TFL*1-like, and *LFY/NDLY* may play important roles in reproductive development in conifers [14], and we analysed their expression patterns in EMCs and LMCs (Fig. 3a). Unfortunately, we did not find any one gene or set of genes that clearly separated the EP and LP samples using principal component analysis (PCA), validating that EPs and LPs do not have strong genomic signatures of significant developmental differences in the early spring. However, based on the mean values of six trees per group, PCA using these genes could distinguish among the different sample groups (Fig. 3b), and it appeared that the EMCs developed slightly faster than the LMCs (Fig. 3b). The Class B genes *DAL11*–*13* [15], class C gene *DAL2* [16], and *DAL1* and *MADS2* were the main *MADS-box* genes that were highly abundant in male cones (Fig. 3a). However, none of these genes were differentially expressed between EMCs and LMCs (Supplemental Figure 1).

As *EBB1* was a key candidate in bud burst regulation in trees [17], we assessed the expression levels of six *EBB1* homologues in *P. tabuliformis*. But we did not identify any significant expression differences between EMCs and LMCs for any of these homologues (Supplemental Figure 2).

### Time-course comparative transcriptome analyses reveal two distinct transcriptional modules underlying male cone development in EPs and LPs

**Fig. 4:**
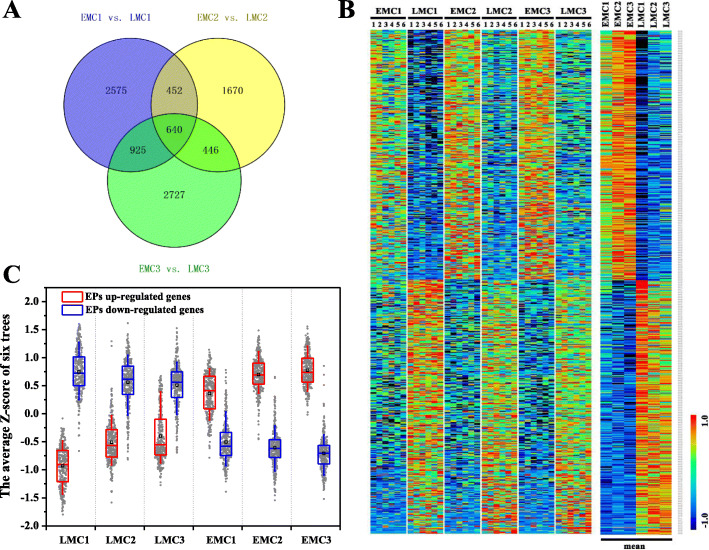
Expression patterns of genes that were stably differentially expressed between EMCs and LMCs at every time point. **a** A total of 640 differentially expressed genes overlapped between EMCs and LMCs at every time point (*P* < 0.05). **b** Heat map of 640 genes in EMCs and LMCs. The data from six biological replicates are shown individually on the left, and shown as averages on the right. The data were normalised by Z-score transformation. **c** The expression pattern shifts of 640 genes during male cone development in the spring. The boxes represent the median and 25th–75th percentiles of the Z-scores of two sets of genes, and the whiskers represent the 10th and 90th percentiles. The grey data points represent the Z-score distribution. The mean Z-score values of six biological replicates were used. The red box and blue box represent genes with higher and lower expression levels in EMCs and LMCs, respectively

To determine whether EMCs and LMCs express distinct transcriptional modules, the differentially expressed genes that overlapped between EMCs and LMCs at every time point were selected and further analysed (Fig. 4a). A total of 640 genes were differentially expressed between EMCs and LMCs, of which 317 were more abundant in EMCs; the other 323 genes were highly expressed in LMCs (Fig. 4b, Supplemental Data Set 1). Interestingly, in both EMCs and LMCs, the expression of upregulated genes in EPs gradually increased over the course of development, whereas the expression of downregulated genes in EPs gradually declined (Fig. 4b, c). This similar trend in expression level shift between the two transcriptional modules suggests that the LP-preferred transcriptomic pattern is actually weakened, whereas the EP-preferred pattern is gradually enhanced, over the course of development (Fig. 4c, Supplemental Data Set 2).

### The EP- and LP-preferred transcriptional modules associated with pollen shedding time are expressed not only in male cones, but are also consistently expressed in needles

**Fig. 5:**
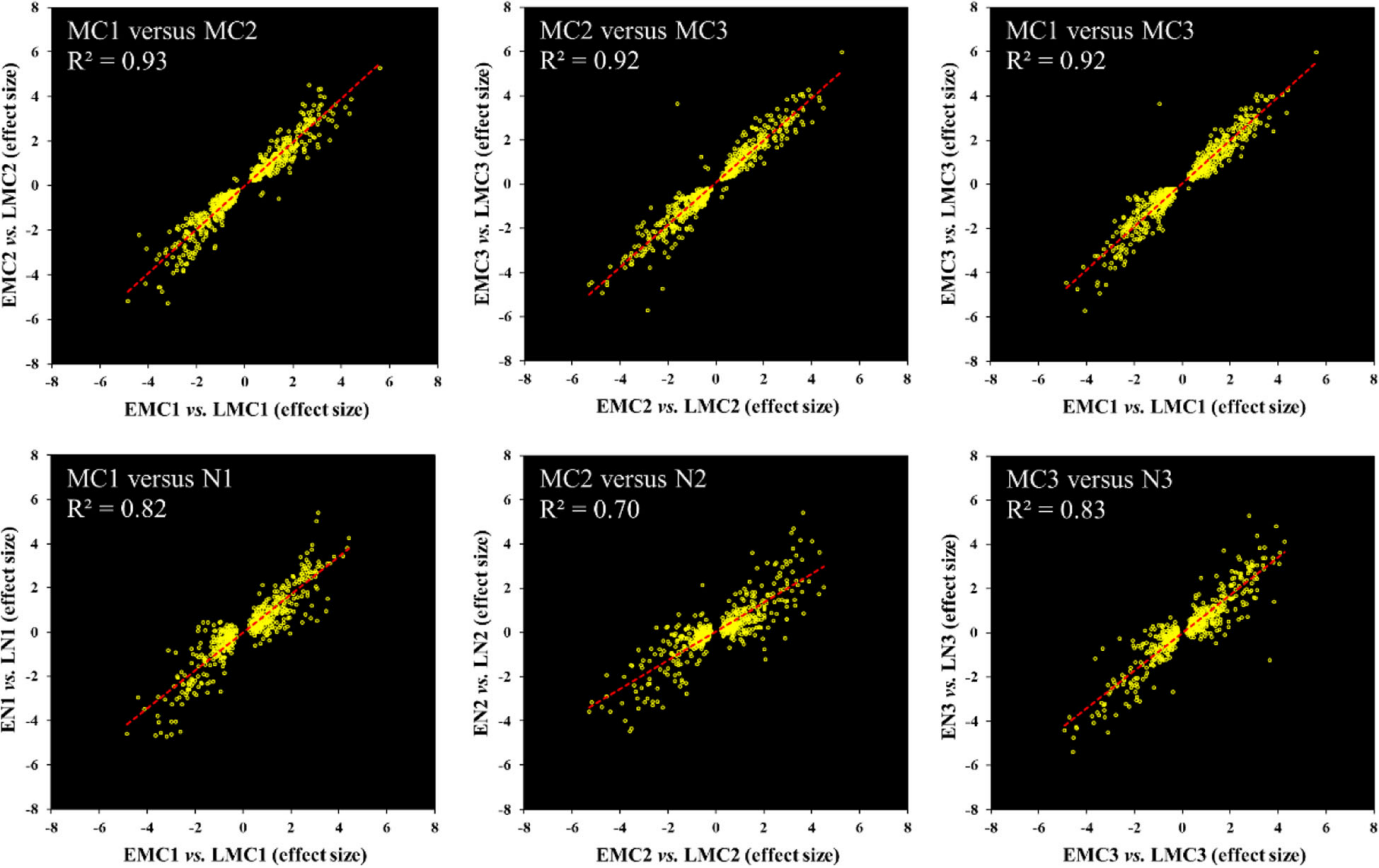
The transcriptional differences between EPs and LPs were consistent in male cones and needles. Scatter plots of effect sizes of EMC/LMC and EN/LN and Pearson correlations indicate that the transcriptional profiles of needles and male cones were strongly correlated. The top panel is based on all 640 genes, and the bottom panel shows the 512 genes that were expressed (mean TPM > 1) in needles. MC, male cones; EMC and LMC, male cones from EPs and LPs, respectively; N, needles; EN and LN, needles from EPs and LPs, respectively. The numerical order indicates the different sampling times

Pines have a distinctly different trait from deciduous trees, such as poplar, they maintain their green foliage throughout the winter. To determine whether distinct transcriptional modules were specifically expressed in male cones, or also synchronously expressed in other tissues such as needles. We analysed the transcriptome of needles that were collected at the same time as male cones from the EPs and LPs. The results showed that 80% (512) of the 640 genes were also expressed in needles; furthermore, the expressional profiles of these genes were highly similar between male cones and needles (Fig. 5). Synchronous with the expressional shift in male cones, the EP-preferred transcriptomic pattern was also gradually improved, and the LP-preferred pattern, weakened over the course of development (Supplemental Figure 3). These results indicate that there is likely a global regulation of both male cones and needles underlying pollen shedding time control in pines.

**Fig. 6:**
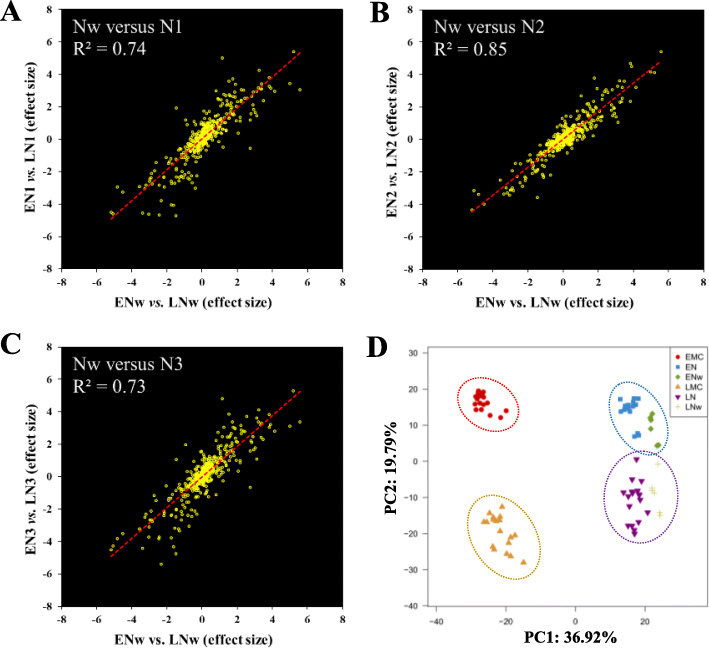
The EP- and LP-preferred expression patterns were consistent even during the winter dormancy period. **a**, **b**, and **c** Pearson correlations of expression differences among needles in mid-winter and spring. Scatter plots show 512 genes expressed (mean TPM > 1) in needles in winter. N, needles; EN and LN, needles from EPs and LPs, respectively. The numerical order indicates the different sampling times. ENw and LNw indicate needles sampled in the winter. **d** PCA based on the expression data of 640 genes. Each symbol represents a sample of individuals; a total of 84 samples are shown in the plot. All of the male cone and needle samples collected either in winter or in spring could be divided into EP (top) and LP (bottom) groups

During the dormancy period in winter, mRNA abundance was significantly decreased at the transcriptome-wide level (Supplemental Figure 4). Surprisingly, we found that the transcriptional module differences between EPs and LPs persisted even in the middle of winter (Fig. 6a–c). There was a strong correlation between the expressional fold-change in the needles in winter and early spring (Fig. 6a–c). Based on the 640 differentially expressed genes screened between EMCs and LMCs, all of the needle samples collected either in winter or spring were clearly divided into EP and LP groups (Fig. 6d). This indicates that the EP- and LP transcriptional modules associated with pollen shedding time were globally consistent.

### The EPs were more sensitive to relatively warmer temperatures than the LPs during the cold winter and early spring

**Fig. 7:**
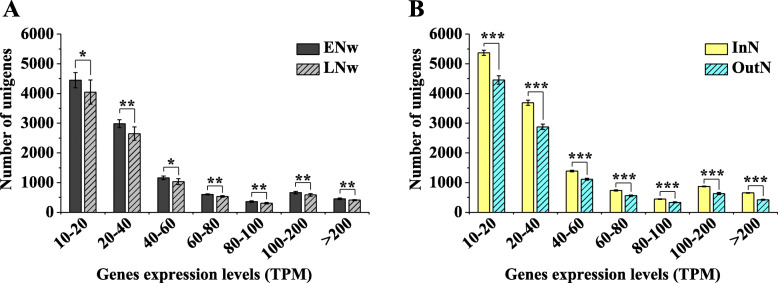
The transcriptomic activity of needles in EP and LP trees kept indoors and outdoors in mid-winter. **a** Frequency distribution of highly abundant genes (TPM > 10) in the needles of EPs and LPs. ENw and LNw indicate the needles of EPs and LPs in mid-winter. *P*-values for significance of differences in the mean (*t*-test) are given above the bars. *P < 0.05, ** *P* < 0.01. Error bars indicate the SE of six biological replicates. **b** Frequency distribution of highly abundant genes (TPM > 10) in needles of trees kept indoors (In) and outdoors (Out) in the mid-winter. The trees were moved indoors for one week before sampling, and maintained in greenhouses under natural light and photoperiod conditions. Temperature was maintained at 8–10 °C during the day and 0–4 °C at night. ***P < 0.001. Error bars indicate SEs of nine biological replicates

Because the EPs and LPs were grown under very similar temperature conditions, it seemed that the EPs required less heat accumulation to induce pollen shedding than the LPs. We speculated that the EPs may be more sensitive to temperature, and particularly to low temperatures that inhibit physiological activities. To test this hypothesis, we collected needles from EPs and LPs at noon on a relatively warm day in the middle of winter (13–2 °C atmospheric temperature). We found higher gene expression levels in EPs than in LPs, indicating that the EPs had higher transcriptomic activity than the LPs (Fig. 7a). However, at relatively high temperatures in the spring this difference in transcriptomic activity between EPs and LPs was no longer noticeable (Supplemental Figure 4). To confirm that transcriptomic activity responds to relatively warm temperatures even during dormancy (trees has already undergone the chilling required and transitioned into ecodormancy), several potted 7-year-old *P. tabuliformis* trees were divided into two equal blocks. One block was moved to a greenhouse, whereas the other was kept outdoors in the middle of winter; the mRNA abundance of the needles of 18 trees was analysed  one week later. The results showed that the trees in the greenhouse had significantly higher transcriptomic activity than the outdoor trees (Fig. 7b).

**Fig. 8:**
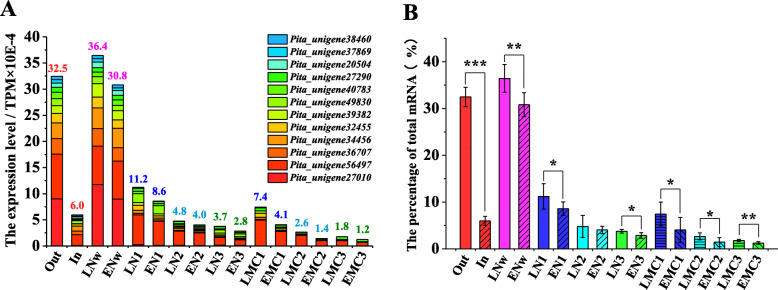
Expression profiles of genes highly accumulated in mid-winter. **a** The top 12 most abundant genes in needles in mid-winter are shown. The numbers above each bar indicate the sum of the abundance of these genes. **b** The percentages of these 12 genes by expression level accounted for the total mRNA. *P < 0.05, ** *P* < 0.01, *** *P* < 0.001. Error bars indicate SEs of at least six biological replicates

At low temperatures, most genes had lower expression levels (Fig. 7b, Supplemental Figure 5). However, a small number of genes accumulated at surprisingly high levels in cold temperatures (Fig. 8a, b), as the top 12 most abundant genes accounted for one-third of the total mRNA in the outdoor trees (Fig. 8b). Interestingly, these top 12 genes were all significantly repressed at relatively warmer temperatures (Fig. 8a, b, Supplemental Figure 6), whereas under the same temperatures, both male cones and needles from EPs had a lower accumulation of these genes than LPs (Fig. 8a, b). These results suggest that the EPs were more sensitive to relatively warmer temperatures than LPs during the cold winter and early spring.

### Expression patterns of EP- and LP-preferred transcriptional modules under different conditions

**Fig. 9:**
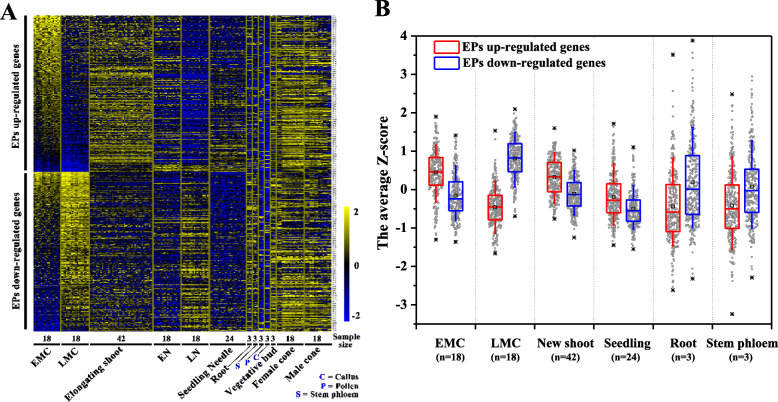
Expression patterns of EP- and LP-preferred transcriptional modules under different conditions. **a** The expression profiles of 640 genes, in a total of 189 *P. tabuliformis* samples under a variety of growth and treatment conditions, are shown in the heat map. The data were normalised by Z-score transformation. EMC and LMC indicate male cones of EPs and LPs, respectively; EN and LN indicate needles of EPs and LPs, respectively; the elongating shoots were sampled from trees of different ages in the spring; seedling needles were sampled from 1-month-old seedlings under different photoperiods, including long day (16 h: 8 h), short day (10 h: 14 h) and control (14 h: 10 h), and different light conditions (red, far red and bright light). Different tissues (needles, roots, pollen, stem phloem, vegetative buds, and developing male and female cones) were sampled from 33-year-old trees in the spring. Callus was induced from hypocotyls. **b** Expression patterns of 640 genes in different tissue types. The mean Z-score values of all biological replicates were used. The boxes display the first quartile, median, and third quartile data, the whiskers range from 10 to 90%, and * indicates the minimum and maximum data. The distribution of the data is represented as a scatter plot. The red box and blue box represent genes with higher and lower expression levels in EMCs than LMCs, respectively

As the EP- and LP-preferred transcriptional modules were expressed in a globally consistent manner, determining their expression patterns under various conditions was the next step in understanding their biological functions. Seedlings were subjected to several treatment and growth conditions, such as different photoperiods including long day (16 h: 8 h), short day (10 h: 14 h) and control (14 h: 10 h); different light conditions (red, far red, and bright light) [18]; and different tissues (needles, root, pollen, stem phloem, callus, vegetative bud, and developing male and female cones from 33-year-old trees [14]); the elongating new spring shoots from trees of different ages were also analysed (Fig. 9a). The results indicated that differences in expression patterns were determined primarily by tissue type rather than treatment conditions (Fig. 9a). These genes were highly expressed in reproductive tissues, whereas they were repressed mainly in pollen and callus tissue (Fig. 9a). We found that the elongating new shoots adopted the preferred pattern of EP; 1-month-old seedlings adopted a similar pattern, while mature root and stem phloem expressed to some extent the preferred pattern of LP (Fig. 9b). These results suggest that the EP-preferred pattern may facilitate rapid cellular proliferation and accelerated growth.

### The EP-preferred transcriptional pattern enhances the nucleic acid synthesis and stress resistance pathways

**Fig. 10:**
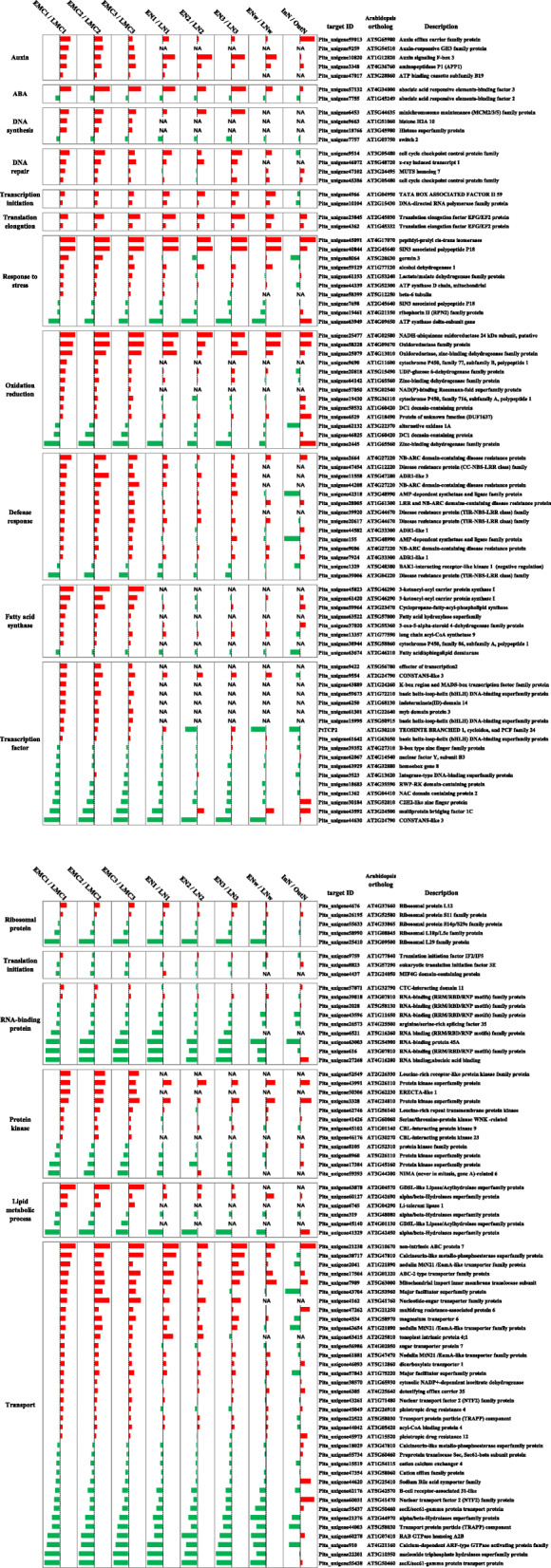
The expression and functional enrichment and annotation of EP- and LP-preferred transcriptional modules. ABA, abscisic acid. The red and green histograms indicate genes that are expressed at higher levels and lower levels, respectively, in EPs or under indoor conditions. InN, needles of indoor trees; OutN, needles of outdoor trees. The height of the bar represents the effect size (analogous to fold-change) values that were obtained using Sleuth software [21]. Values represent the means of at least six biological replicates

Functional annotations remain inaccessible for many reported genes in conifer [19]; thus, only 58% (369) of the genes were evaluated for functional annotations. However, we found a strong genomic signature of enhanced nucleic acid synthesis and stress resistance in the EP-preferred transcriptional pattern (Fig. 10). The activation of DNA repair, DNA synthesis, and RNA synthesis pathways (Supplemental Figure 7) were highlighted by MapMan profiling tools [20]. Among all of these genes with functional annotations associated with DNA synthesis, DNA repair, transcription initiation, and translation elongation, 11 were upregulated in EPs, and only 1 gene was repressed (Fig. 10). Additionally, other genes were associated with stress response, oxidation/reduction, and defence response were enriched in the EP-preferred module (Fig. 10). These results highlighted that the EP-preferred module facilitates rapid growth and retains higher transcriptional activity at low temperatures. Unexpectedly, only a few genes were functionally related to phytohormones, revealing that the auxin signalling pathway in EPs was enhanced (Fig. 10). Some transcription factors were regulated in EPs and LPs, but interestingly, most of the ones upregulated in EPs were specifically expressed in male cones (Fig. 10). Additionally, the gene ontology (GO) term “active transmembrane transporter activity” was the most enriched, and we found 36 genes involved in transport processes differentially regulated in EPs and LPs; 22 were upregulated in EPs and the rest upregulated in LPs (Fig. 10). The transport activity regulation was highly consistent between male cones and needles in EPs and LPs but differed from the warmer temperature response (Fig. 10), indicating that the regulation of transportation is a proactive process rather than simply response to temperature.

## Discussion

Seasonal flowering time is a critical trait that mainly determines reproductive success in temperate tree species. This developmental process is strictly regulated by both environmental signals and endogenous cues. Although the molecular basis of flowering time regulation in trees remains largely unknown, ample observations provide strong evidence, albeit correlative, that temperature plays an important role in this developmental process. In the *P. tabuliformis* seed orchard, although the pollen shedding date fluctuated with the temperature during spring, the selected EPs always shed their pollen approximately one week earlier than neighbouring LPs every spring (Fig. 1), indicating that this process was under strict genetic control. Because the male cone primordia are formed in the autumn of the previous year, the difference in pollen shedding time may because male cones were already in a different developmental stage after months of dormancy, or because the male cones of EPs may take a shorter time to achieve pollen shedding than those of LPs. It is difficult to identify morphological differences in male cones at very similar developmental stages in early spring. Based on time-course comparative transcriptome analyses, we found that there were indeed some differentially expressed genes between EMCs and LMCs, even though much less than the expressional change caused by 5–10 days of development (Fig. 2). A previous observation showed that moving pines with earlier-formed male cones from a greenhouse to the outdoors did not result in earlier pollen shedding [22]. The inhibition of pollen shedding by earlier-developed male cones is crucial for controlling the flowering date to coincide with favourable environmental conditions. This suggests that pollen shedding time is under very strict genetic control under certain growth conditions.

In *Arabidopsis*, the *FLOWERING LOCUS T* (*FT*) gene has been identified as a key integrator of environmental signals (photoperiod and temperature) and endogenous cues (age and gibberellins, GA) for the regulation of flowering time [3]. The *FT* orthologues in *Populus* [23, 24] and apple [25] are also involved in regulating the multi-year delay in first-time flowering. The *FT* orthologues in poplar likely to be play a role in seasonal flowering time regulation, as the *PtFT1* was found to control growth cessation and bud set during the autumn [24], whereas *PdFT2* expression was associated with seasonal flower initiation [23]. Thus, further investigations are needed to unravel the genetic regulation of seasonal flowering time during the annual growth cycle. However, whole genome sequencing revealed that the absence of orthologues of *FT* genes [26], and all of the *FT/TFL1*-like genes in conifers act as flowering repressors when heterologously expressed in *Arabidopsis*, and play a role in growth rhythm and bud set regulation [27–32]. As transgenic analysis is very difficult and not yet possible for most conifer trees, the effects of functional defects of these repressors remain unclear. Although this would be hypothetical, other genes in conifers are likely to act as the “florigen”. Evidence suggests that some *MADS-box* genes are candidate key activators in the transition from juvenile-to-adult in conifers [33, 34], however, their exact roles in both first flowering and seasonal flowering time regulation remain to be explore.

The *MADS-box* gene family members, which play fundamental role in plant reproductive development, were differentially expressed according to the developmental stages of male and female cones in pines [14]. We did not find any *MADS-box* genes that were stably differentially expressed between EMCs and LMCs, although we observed a trend whereby the EMCs developed slightly earlier than the LMCs (Fig. 3). Therefore, our data supports that male cones of EPs and LPs do not have strong genomic signatures of significant developmental differences in early spring.

Although the molecular regulation of the seasonal flowering time in temperate forest trees remains largely unknown, extensive studies have been conducted on the dormancy release and vegetative buds burst in spring [19, 35]. The *EARLY BUD-BREAK 1* (*EBB1*) gene was first identified in poplar, and it work as a positive regulator in bud burst [36], and the function of *EBB1* is likely conserved across a wide range of woody perennials, including conifer trees [17]. Interestingly, *EBB1* also noticed similar regulatory functions in flower bud break in the Japanese pear [37]. Although its expression peaks only shortly before flower bud break and is not associated with the flowering stage, early bud burst may result in early flowering. We identified three *EBB1* orthologous gene in *P. tabuliformis*, however, we did not find any one was differentially expressed between EMCs and LMCs (Supplemental Figure 2).

An earlier observation showed that moving pines with earlier male cones from a greenhouse to the outside did not cause earlier pollen shedding [22]. To coincide with favourable environmental conditions, the inhibition of pollen shedding by earlier developed male cones is crucial for controlling the flowering date. This suggests that under certain conditions of growth the time of pollen shedding is under very strict genetic control. In the present study, we found that two distinct transcriptional modules comprising a total of 640 genes were differentially expressed between EMCs and LMCs (Fig. 4a); this difference was not only specific to male cones, but was also consistent among the needles (Fig. 5), suggesting that expressional differences were not due to different developmental stages between EMCs and LMCs. Interestingly, in both male cones and needles, the abundance of the EP-preferred transcriptional module gradually increased with male cone development in both EPs and LPs, whereas the LP-preferred module gradually declined (Fig. 4b, c; Supplemental Figure 3). Thus, the EP-preferred transcriptomic pattern conformed more closely with male cone developmental trends than the LP-preferred pattern.

Unexpectedly, the EP- and LP-preferred transcriptional patterns were not only present in male cones, but were also consistently expressed in needles, even the same pattern was observed during the dormancy period (Figs. 5 and 6). Although the gene expression levels were significantly affected by the tissue type and prevailing growth conditions, the expression fold-change between the EPs and LPs was always strongly linearly related (Figs. 5 and 6), indicating that the two different transcriptional patterns were the genomic signatures of EPs and LPs. It is possible to regulate other developmental processes besides the pollen-shedding date. We observed EPs showed higher transcriptomic activity during the winter season (Fig. 7a) because the transcriptomic activity was very sensitive to relatively warmer temperatures (Fig. 7b), indicating that the EPs were probably more sensitive to relatively warmer temperatures than LPs during the cold winter and early spring. The *Pita_unigene27010* encoding an EARLY LIGHT-INDUCABLE PROTEIN (ELIP) was the most abundant gene in the needles in the winter, accounting for almost 10% of the total mRNA (Fig. 8a). The *ELIP* genes were first identified as early response genes under light stress, just as the name implies [38]; however, its function in light stress response is still not well understood [39]. In *Arabidopsis*, *ELIP* was later found to also be induced by chilling [40]. Interestingly, *ELIP* was also one of the most abundantly expressed genes (the 12th-highest) in the dormancy buds of poplar during mid-winter, and was suddenly downregulated in May [41]. *ELIP* was probably very sensitive to cold temperatures in poplar, because complete dormancy appeared insufficient to induce the observed extremely high *EILP* expression [42]. In pine, *ELIP* has been shown to be the most rapidly and highly cold-inducible gene [43]. In the present study, we also found *PtELIP* (GenBank: JQ071215) was very sensitive to warmer temperatures (Fig. 8a); thus, it can be used as a good temperature response-sensitive candidate markers in trees. Therefore, a higher *PtELIP* expression level in LPs than Eps, in both male cones and needles at all time points, indicates that the EPs were more sensitive to relatively warmer temperatures than LPs during the cold winter and early spring. In addition, many stress response genes were enriched in the EP-preferred module (Fig. 10), which may facilitate the maintenance of higher transcriptional activity at low temperatures in EPs than in LPs. The lower physiological activity temperature threshold means that the EPs require less heat accumulation to induce pollen shedding than LPs.

The identification of specific gene functions during the development of conifers is very difficult, and in many species, it is not yet possible. To uncover the possible roles of the EP- and LP-preferred modules in the growth or environmental response of pines, we analysed the expression patterns of these genes under different growth and treatment conditions. Photoperiod, light quality, and age did not appear to be involved in the establishment of differential expression patterns, whereas the main differences likely associated with different growth stages of cells in different tissues, rather than with tissue types per se. The EP-preferred pattern was adopted by rapidly growing tissues, such as new elongating shoots in the spring and 1-month-old seedlings, suggesting that it may have growth-promoting functions. Genes with functions related to DNA synthesis, DNA repair, transcription initiation, and translation elongation exhibited higher activity levels in the EP-preferred pattern (Fig. 10), consistent with the growth-activating function.

Unexpectedly, only a few genes functionally related to phytohormones were found in both EP- and LP-preferred modules, and these genes were mainly related to auxin. In contrast, a large number of previous studies have elucidated the important roles of phytohormones in flowering induction in *Arabidopsis*. Over the past half century, multiple phytohormones and plant growth regulators have been applied for inducing or enhancing cone flowering in conifers and showed a variety of regulatory effects [44]. Phytohormones are also involved in apical bud formation and dormancy induction. Time-course comparative transcriptome analyses of the apex in poplar trees showed that GA signalling was repressed, and ET and ABA signalling triggered, during this process [42]. GA4 was shown to induce bud burst through enhancing the 1, 3-beta-glucanase genes in poplar [45]. Interestingly, no effects of auxin were seen during the dormancy induction process in poplar [45], whereas in conifer trees, several phytohormones (IAA, CKs, ABA, and their metabolites) were quantified during the growing season in Douglas fir (*Pseudotsuga menziesii*), and only auxin showed at peak during the rapid elongation stage of shoots; moreover, auxin was also the only phytohormone with a trend that correlated with cone productivity [46]. It is noteworthy that the rapidly elongating new shoots also adopted the EP-preferred module (Fig. 9), and appeared to correspond with all auxin-related genes activated in the present study module (Fig. 10).

## Conclusions

The present study provides new insights about the strict genetic control of pollen shedding time in pine. Time-course comparative transcriptome analyses reveals that early pollen shedding trees were more sensitive to relatively warmer temperature than late pollen shedding trees during the cold winter and early spring. A set of 640 genes were identified that differently expressed in male cones between two group trees. The EPs preferred pattern enhanced the nucleic acid synthesis and stress resistance pathways that may facilitate the rapid cell proliferation and fast growth. In addition, these data firstly indicating that the pollen shedding time control in pine probably underlying a global regulation from both male cones and needles. Our results provide new insights about the molecular mechanisms of seasonal flowering time regulation in pines, and will helpful in related studies in the future.

## Methods

### Plant material and growth conditions

EPs and LPs samples of *Pinus tabuliformis* Carr. were collected from a total of 217 plus-tree clones which were selected from natural populations in a primary clonal seed orchard located in Pingquan City, Hebei Province, China (118°44.6758′ E, 40°98.8784′ N, 560–580 m above sea level). These selected clones were grafted on 2-year-old rootstocks in 1984 and then transplanted into the orchard during the flowering year. In the seed orchard, the daily average maximum/minimum temperatures in January and March were − 2 °C/− 14 °C and 10 °C/− 4 °C, respectively, and the average precipitation was 1 mm and 5 mm in January and March, respectively. The temperature was maintained at 8–10 °C during the day and 0–4 °C at night, and natural light and photoperiod conditions were utilized in greenhouses.

All of the clones were evenly divided into three groups: the early-pollen shedding group, the middle group, and the late-pollen shedding group, according to the flowering dates recorded over the last 3 years. Six early-pollen shedding trees (EPs) and six late-pollen shedding trees (LPs) which planted near each other with non-overlapping pollen shedding peaks were selected to compare with each other. Male cone buds were collected from three south-side shoots of each EP and LP at 5-day intervals beginningfrom January 8 to March 20, and the six needles closest to the selected male cones were also sampled simultaneously (Fig. 1). The samples were quickly frozen in liquid nitrogen after collection and stored at − 80 °C in the laboratory for further analysis.

### RNA-seq analysis

Total RNA quantity and purity were evaluated using a Nano Photometer spectrophotometer (Implen, Westlake Village, CA, USA), and RNA concentration was measured by a Qubit RNA Assay kit and Qubit 2.0 Fluorometer (Life Technologies, Carlsbad, CA, USA). The RNA Nano 6000 Assay Kit of the Bioanalyzer 2100 system (Agilent Technologies, Santa Clara, CA, USA) was used to assess RNA integrity. The mRNA was fragmented into small pieces using divalent cations at high temperatures. The final complementary DNA (cDNA) library was created using cleaved RNA fragments which were reverse-transcribed according to the protocol provided by the mRNA-Seq sample preparation kit (Illumina, Inc., San Diego, CA, USA). The average size of insertion for the paired-end libraries was 200–300 base pairs (bp). The paired-end module was used to sequence the pooled libraries on the Illumina Hiseq X Ten platform (2 × 150 bp). The clean reads were mapped to the *P. tabuliformis* reference transcriptome [47], and the transcript abundances estimation was estimated using Kallisto (0.44) software [48]. Sleuth (0.28) software was used for differential expression analysis [21]. The different gene expression patterns among samples and conditions were calculated and displayed based on data normalized by Z-score transformation [49].

## Supplementary information

**Additional file 1: Figure S1.** Expression profiles of genes highly abundant in male cones from early pollen-shedding trees (EPs) and late pollen-shedding trees (LPs).

**Additional file 2: Figure S2.** Expression levels of *Pinus tabuliformis EBB1* homologues in male cones from early pollen-shedding trees (EPs) and late pollen-shedding trees (LPs).

**Additional file 3: Figure S3.** EP- and LP-preferred transcriptomic pattern shifts during male cone development.

**Additional file 4: Figure S4.** Comparison of transcriptomic activity between male cones and needles of EPs and LPs in the spring.

**Additional file 5: Figure S5.** mRNA accumulation was significantly decreased at the transcriptome-wide level in the winter.

**Additional file 6: Figure S6.** Comparison of the expression profiles of the top 12 most abundant genes in mid-winter between male cones and needles from EPs and LPs.

**Additional file 7: Figure S7.** MapMan cell function overview maps showing differences between EPs and LPs.

**Additional file 8: Data Set 1.** Expression levels of 640 genes in all analysed samples.

**Additional file 9: Data Set 2.** Normalised Z-scores of 640 genes in male cones of EPs and LPs.

**Additional file 10: Data Set 3.** Functional annotations of the two modules.

**Additional file 11: Data Set 4.** Normalised Z-score values of 640 genes in all analysed samples.

**Additional file 12: Data Set 5.** The sequences of all 640 transcripts.

**Additional file 13: Data Set 6.** The NCBI accession number and library IDs of the RNA-seq data.

## Data Availability

The expression level (TPM), Z-score normalized value between EMC and LMC, the detailed functional annotation and *p* values, Z-score normalized value between different growth conditions were provide in Supplemental Data Set 1–4, respectively. The sequences of all the 640 transcripts were provide in Supplemental Data Set 5. All of the RNA-Seq raw data that support the findings of this study are available in the NCBI Sequence Read Archive. The accession number and the corresponding library ID used in this study are provided in Supplementary Data Set 6. The datasets used and/or analyzed during the current study available from the corresponding author on reasonable request.

## Abbreviations

EPs: Early pollen shedding trees
LPs: Late pollen shedding trees
TF: Transcription factor
EN: Needles of EPs
LN: Needles of LPs
EMC: Male cones of EPs
LMC: Male cones of LPs
OutN: Needles of outdoor trees
InN: Needles of indoor trees
GA: Gibberellins
CK: Cytokines
ABA: Abscisic acid
ET: Ethylene
JA: Jasmonic acid
SA: Salicylic acid
