# Supplementary material for: Comparative transcriptome analyses reveal two distinct transcriptional modules associated with pollen shedding time in pine
Source: BMC Genomics. 2020 Jul 22;21:504. doi: 10.1186/s12864-020-06880-9 (PMC7374968; doi:10.1186/s12864-020-06880-9)
Supplement: Supplementary file 1 — Additional file 1: Figure S1. Expression profiles of genes highly abundant in male cones from early pollen-shedding trees (EPs) and late pollen-shedding trees (LPs). [file 12864_2020_6880_MOESM1_ESM.docx]

**

**

**
