# Supplementary figures and images for: Comparative transcriptome analyses reveal two distinct transcriptional modules associated with pollen shedding time in pine
Source: BMC Genomics. 2020 Jul 22;21:504. doi: 10.1186/s12864-020-06880-9 (PMC7374968; doi:10.1186/s12864-020-06880-9)

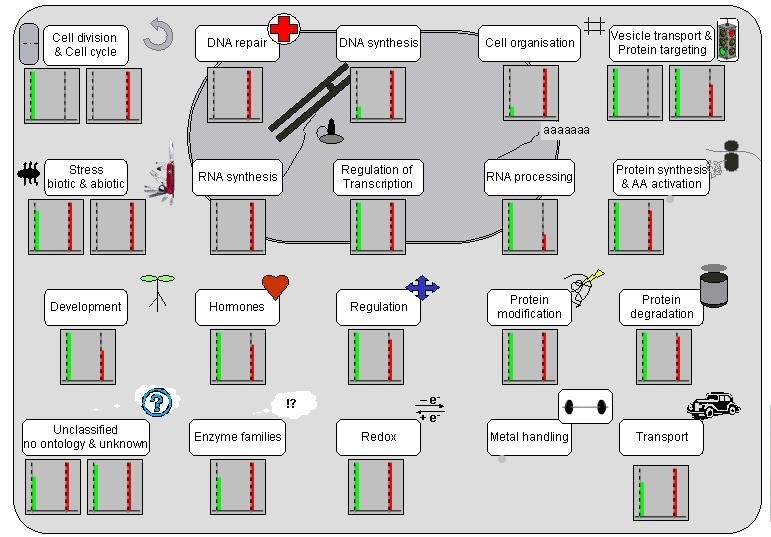

Supplement: Supplementary file 7 — Additional file 7: Figure S7. MapMan cell function overview maps showing differences between EPs and LPs. [file 12864_2020_6880_MOESM7_ESM.jpg]
