# Supplementary material for: Comparative transcriptome analyses reveal two distinct transcriptional modules associated with pollen shedding time in pine
Source: BMC Genomics. 2020 Jul 22;21:504. doi: 10.1186/s12864-020-06880-9 (PMC7374968; doi:10.1186/s12864-020-06880-9)
Supplement: Supplementary file 3 [file 12864_2020_6880_MOESM2_ESM.docx]

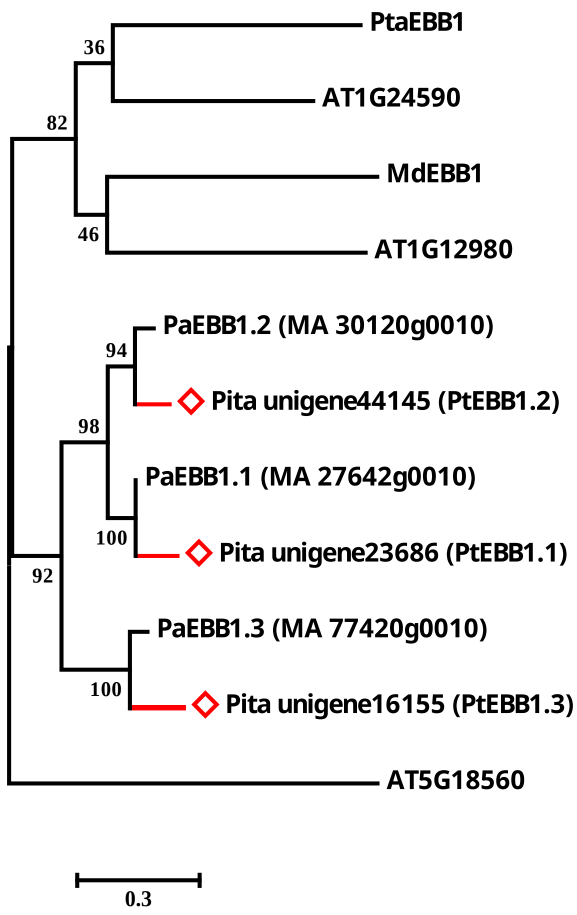
PtaEBB1
AT1G24590
MdEBB1
AT1G12980
PaEBB1.2 (MA 30120g0010)
Pita unigene44145 (PtEBB1.2)
PaEBB1.1 (MA 27642g0010)
Pita unigene23686 (PtEBB1.1)
PaEBB1.3 (MA 77420g0010)
Pita unigene16155 (PtEBB1.3)
AT5G18560
36
82
46
94
98
100
92
100
0.3

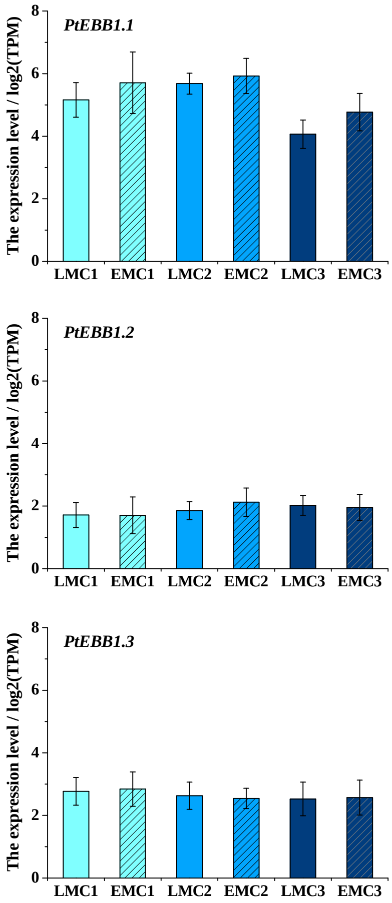
PtEBB1.1
PtEBB1.2
PtEBB1.3
The expression level / log2(TPM)
0
2
4
6
8
LMC1
EMC1
LMC2
EMC2
LMC3
EMC3
