# Supplementary material for: Comparative transcriptome analyses reveal two distinct transcriptional modules associated with pollen shedding time in pine
Source: BMC Genomics. 2020 Jul 22;21:504. doi: 10.1186/s12864-020-06880-9 (PMC7374968; doi:10.1186/s12864-020-06880-9)
Supplement: Supplementary file 4 [file 12864_2020_6880_MOESM3_ESM.docx]

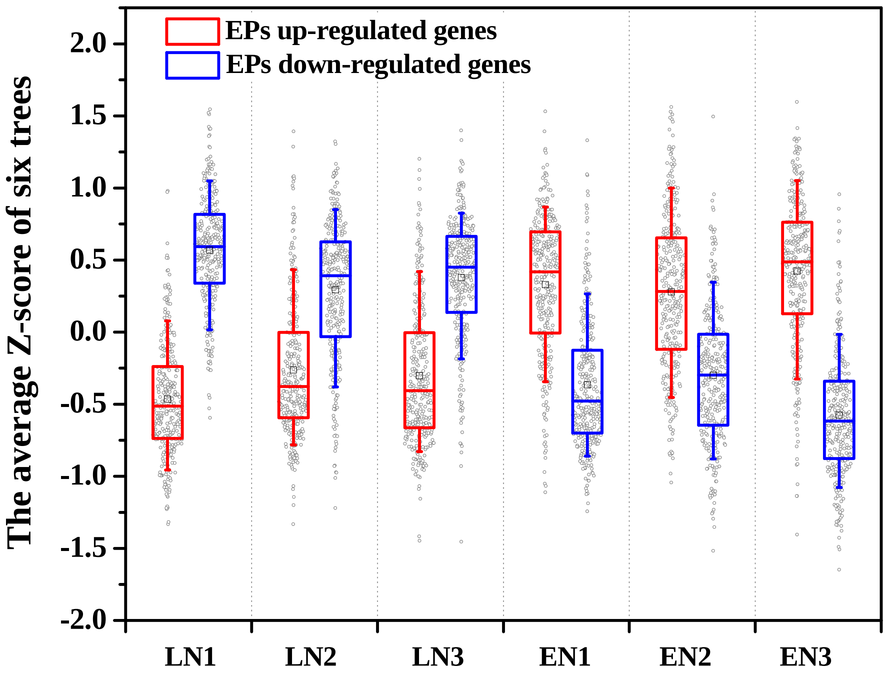

EPs up-regulated genes
EPs down-regulated genes
The average Z-score of six trees
2.0
1.5
1.0
0.5
0.0
-0.5
-1.0
-1.5
-2.0
LN1
LN2
LN3
EN1
EN2
EN3
