# Supplementary material for: Comparative transcriptome analyses reveal two distinct transcriptional modules associated with pollen shedding time in pine
Source: BMC Genomics. 2020 Jul 22;21:504. doi: 10.1186/s12864-020-06880-9 (PMC7374968; doi:10.1186/s12864-020-06880-9)
Supplement: Supplementary file 5 [file 12864_2020_6880_MOESM4_ESM.docx]

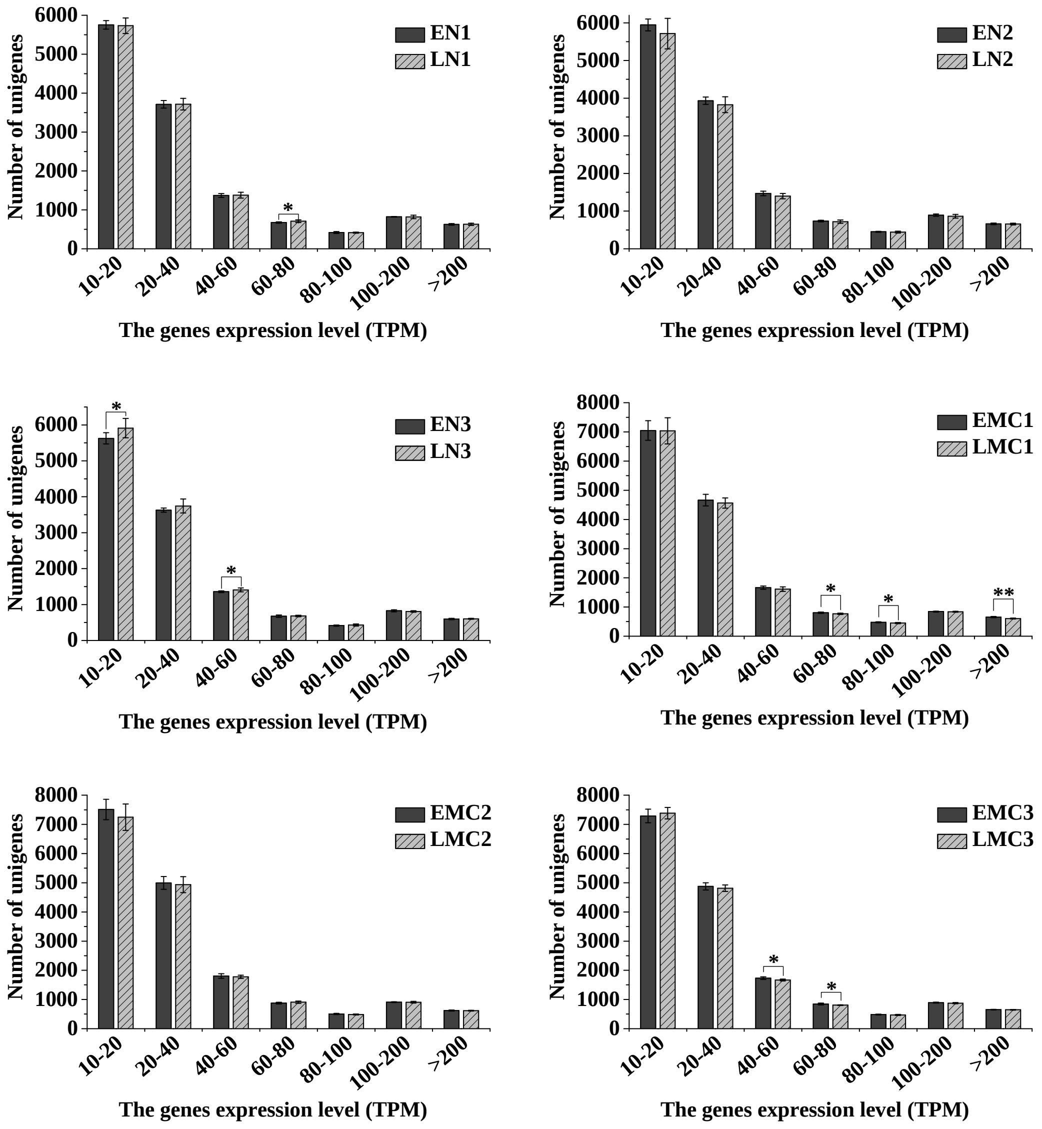

Number of unigenes
The genes expression level (TPM)
10-20
20-40
40-60
60-80
80-100
100-200
>200
EN1
LN1
EN2
LN2
EN3
LN3
EMC1
LMC1
EMC2
LMC2
EMC3
LMC3
0
1000
2000
3000
4000
5000
6000
7000
8000
*
**
