# Supplementary material for: Comparative transcriptome analyses reveal two distinct transcriptional modules associated with pollen shedding time in pine
Source: BMC Genomics. 2020 Jul 22;21:504. doi: 10.1186/s12864-020-06880-9 (PMC7374968; doi:10.1186/s12864-020-06880-9)
Supplement: Supplementary file 6 [file 12864_2020_6880_MOESM5_ESM.docx]

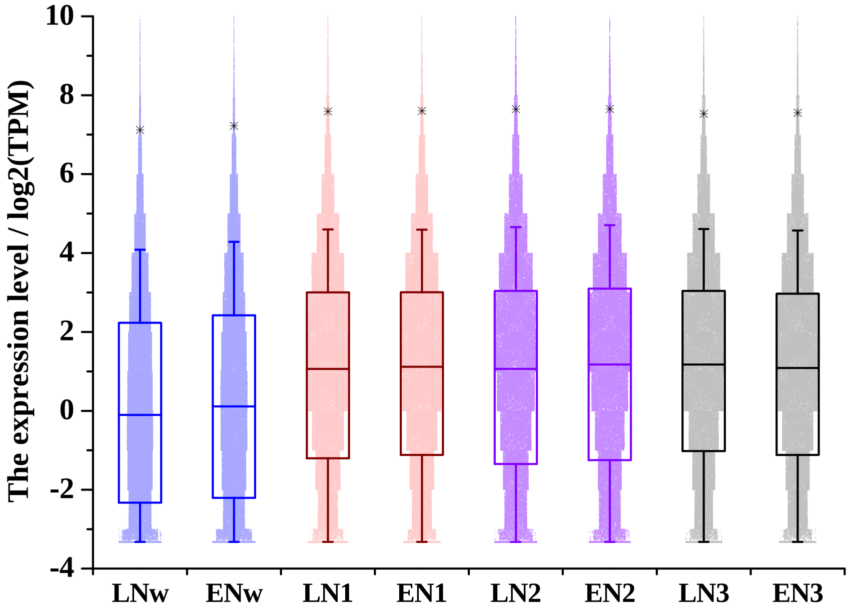

The expression level / log2(TPM)
10
8
6
4
2
0
-2
-4
LNw
ENw
LN1
EN1
LN2
EN2
LN3
EN3
