# Supplementary material for: Comparative transcriptome analyses reveal two distinct transcriptional modules associated with pollen shedding time in pine
Source: BMC Genomics. 2020 Jul 22;21:504. doi: 10.1186/s12864-020-06880-9 (PMC7374968; doi:10.1186/s12864-020-06880-9)
Supplement: Supplementary file 7 [file 12864_2020_6880_MOESM6_ESM.docx]

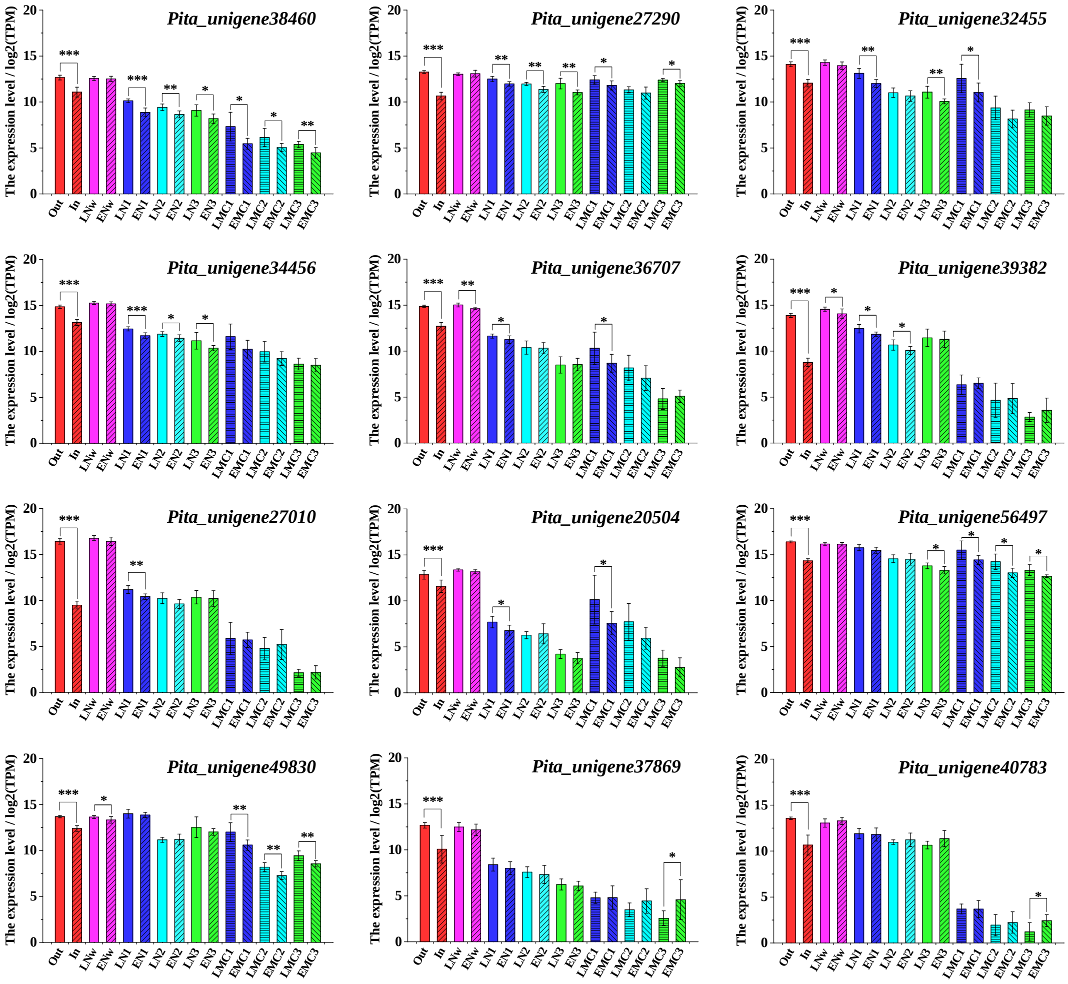
Pita_unigene38460
Pita_unigene27290
Pita_unigene32455
Pita_unigene34456
Pita_unigene36707
Pita_unigene39382
Pita_unigene27010
Pita_unigene20504
Pita_unigene56497
Pita_unigene49830
Pita_unigene37869
Pita_unigene40783
The expression level / log2(TPM)
0
5
10
15
20
Out
In
LNw
ENw
LN1
EN1
LN2
EN2
LN3
EN3
LMC1
EMC1
LMC2
EMC2
LMC3
EMC3
